# Interpreting global trends in type 2 diabetes complications and mortality

**DOI:** 10.1007/s00125-021-05585-2

**Published:** 2021-11-27

**Authors:** Mohammed K. Ali, Jonathan Pearson-Stuttard, Elizabeth Selvin, Edward W. Gregg

**Affiliations:** 1 Hubert Department of Global Health, Rollins School of Public Health, Emory University, Atlanta, GA, USA; 2 Department of Family and Preventive Medicine, School of Medicine, Emory University, Atlanta, GA, USA; 3 Department of Epidemiology and Biostatistics, School of Public Health, Imperial College, London, UK; 4 Health Analytics, Lane Clark & Peacock LLP, London, UK; 5 Department of Epidemiology, Johns Hopkins Bloomberg School of Public Health, Baltimore, MD, USA

**Keywords:** Data quality, Diabetes complications, High-income countries, Low- and middle-income countries, Mortality, Review, Trends

## Abstract

International trends in traditional diabetes complications (cardiovascular, renal, peripheral vascular, ophthalmic, hepatic or neurological diseases) and mortality rates are poorly characterised. An earlier review of studies published up to 2015 demonstrated that most data come from a dozen high-income countries (HICs) in North America, Europe or the Asia–Pacific region and that, in these countries at least, rates of acute glycaemic fluctuations needing medical attention and amputations, myocardial infarction and mortality were all declining over the period. Here, we provide an updated review of published literature on trends in type 2 diabetes complications and mortality in adults since 2015. We also discuss issues related to data collection, analysis and reporting that have influenced global trends in type 2 diabetes and its complications. We found that most data on trends in type 2 diabetes, its complications and mortality come from a small number of HICs with comprehensive surveillance systems, though at least some low- and middle-income countries (LMICs) from Africa and Latin America are represented in this review. The published data suggest that HICs have experienced declines in cardiovascular complication rates and all-cause mortality in people with diabetes. In parallel, cardiovascular complications and mortality rates in people with diabetes have increased over time in LMICs. However, caution is warranted in interpreting trends from LMICs due to extremely sparse data or data that are not comparable across countries. We noted that approaches to case ascertainment and definitions of complications and mortality (numerators) and type 2 diabetes (the denominator) vary widely and influence the interpretation of international data. We offer four key recommendations to more rigorously document trends in rates of type 2 diabetes complications and mortality, over time and worldwide: (1) increasing investments in data collection systems; (2) standardising case definitions and approaches to ascertainment; (3) strengthening analytical capacity; and (4) developing and implementing structured guidelines for reporting of data.

## Introduction

Over the past four decades, the number of adults living with diabetes mellitus worldwide has increased fourfold from 108 million in 1980 to 463 million in 2019 [1, 2]. An estimated 90–95% of these cases are type 2 diabetes. Both type 1 and type 2 diabetes have far-reaching effects on the health and economies of communities. Quantifying how type 2 diabetes complications and mortality rates have changed is an important aspect of monitoring the impact of diabetes and potential policy, programmes and clinical interventions being implemented worldwide. However, data regarding international trends in type 2 diabetes-related complications and mortality are scarce. A previous review aggregated data published up to 2015 on cardiovascular, renal, ophthalmic and acute complications and documented the scarcity of data, describing trends observed in the few countries where published data were available [3].

Here, we compiled the published literature to assess whether newer and more widespread data have emerged since 2015 and what the literature showed. We examine and offer perspectives on a range of potential methodological variations and biases that may explain the observed international trends in type 2 diabetes complications and mortality. Our review focuses on adults with type 2 diabetes, as the pathophysiology, age of onset and progression for type 1 diabetes are quite distinct. We discuss interpretations of these data and propose recommendations that could help improve and harmonise our understanding of the impacts of type 2 diabetes within and across countries.

## Global diabetes complications and mortality: the current evidence

We searched the National Library of Medicine database in March 2021 for articles that reported trends in rates of diabetes-related complications or mortality in adults across different geographical regions. We included articles that reported data with international, national or subnational coverage. We included studies with high-quality data sources such as registries or discharge records and excluded purely modelled estimates [4, 5]. We extracted information and tabulated the countries that were represented, definitions of the denominators and numerators used and a summary of the direction of trends.

The included studies were published during 2015–2021 and were from relatively few countries, the most common being high-income countries (HICs) in Europe, North America and Australasia. The few low- and middle-income countries (LMICs) represented were Ghana and South Africa (Africa), Brazil, Colombia and Argentina (Latin America) and China (Asia). Two articles used the WHO mortality database [6, 7] from approximately 108 countries to report on all-cause and cause-specific mortality trends over 15 years, providing a more global perspective. Most of the studies did not distinguish between diabetes type (type 1, type 2 or other forms of diabetes). The data were generally limited to age- and sex-specific rates, with few, if any, studies examining trends in rates by socioeconomic status. Very few studies from countries with diverse populations provided results according to race/ethnicity.

Data for rates of diabetes complications (Table 1) published between 2016–2021 were predominantly from HICs. Patterns show declining rates of hospitalisation for vascular complications and acute glycaemic fluctuations over time, especially in the decade after 2005. Lower-extremity amputations increased over this period, driven by toe and minor amputations, with declines in major and higher limb amputations. The only middle-income country represented was Brazil, where diabetes-related hospitalisations in the overall population increased over 2008–2019. There were no data on diabetic kidney disease or ophthalmic, hepatic or neurological diseases over time.

Mortality trends in diabetes are typically documented using two methods: (1) population-based sources where cause-specific mortality rates are estimated among people with diabetes who participated in population registries or other administrative datasets or cohort studies and (2) estimates of deaths in the general population where diabetes was recorded on death certificates as the underlying cause or a contributing cause (Table 2). Global data from the WHO [6, 7] and data from some LMICs [8–10] were derived from death certificates, and these showed increasing mortality due to diabetes over time. Meanwhile, data from HICs mostly concerned mortality rates in people with diabetes from population-based sources (e.g. registries or administrative data sources), and these tended to show declines, especially after 2000. Other findings from HICs included less-pronounced declines in mortality rates in adults under the age of 45 years [11, 12], more marked declines in cardiovascular causes of death and increased or stable death rates due to infectious causes such as pneumonia [13, 14].

In countries with diverse race/ethnic populations, an extensive literature has documented how underrepresented race/ethnic or indigenous groups tend to experience higher rates and disproportionate burdens of diabetes complications and mortality [15]. In our review, we noted persistently higher mortality rates in Māori New Zealanders than in their white counterparts [16]. Certain race/ethnic groups are also more likely to experience adverse socioeconomic circumstances and barriers to healthcare access. Some ethnic groups, especially Pacific Islanders and Native Americans, appear to have physiologically higher risk of type 2 diabetes and renal complications [17], but these groups represent very small segments of national datasets, meaning the estimates are imprecise. This suggests that studies dedicated to understanding biological risk are necessary to improve our efforts to address diabetes globally.

The data in our review are similar to those in the previous review, which included data published through 2015 [3]; however, our study had notably fewer data from Scandinavia. Our findings also align with other literature showing that as rates of macrovascular complications and mortality have declined over the past three decades [3, 18], there have been increases in complications such as cancer, dementia, infection, tuberculosis and tropical diseases [3, 11, 19, 20]. Since the largest declines in vascular complications have been observed for older adults, these emerging complications have been more closely observed in this age group. For younger groups, cardiometabolic risk profiles have not improved in HICs [21, 22] or LMICs [23], and previous data show that the rates of vascular complications did not decline as much in younger adults as in middle- or older-age adults [18]. High and increasing diabetes complications and mortality rates observed also align with data showing major gaps in care for adults with diabetes in LMICs [23, 24]. Newer data in this review did not explore whether changes in rates of vascular complications differ by age group or whether these remain the dominant outcomes observed in this younger adult population subgroup. There could also be other reasons for the observed trends, which we discuss below.

## How definitions of denominators can influence rates

### Complications

The denominators of published complications rates in people with type 2 diabetes are generally from population-based data on adults with diagnosed diabetes. Several factors could have influenced these rates.

Changes in diagnostic thresholds for type 2 diabetes may have influenced the pool of adults with diagnosed diabetes. In 1997, the ADA lowered the threshold for diagnosis of diabetes from a fasting glucose of 7.8 mmol/l (140 mg/dl) to 7.0 mmol/l (126 mg/dl) [25]. This lower threshold may mean that the characteristics of people with newly diagnosed diabetes have changed over time. In other words, sociodemographic and clinical characteristics of adults with type 2 diabetes today differ from those several decades ago. For example, in HICs, higher proportions of today’s adults with diabetes represent different race/ethnic groups, lower socioeconomic classes and higher obesity segments than in decades past.

The use of HbA_1c_ for diagnosis of diabetes could have also influenced trends. Recommended by the ADA in 2010 [26, 27], HbA_1c_ was adopted as a diagnostic test by the WHO and other major diabetes organisations across the globe. HbA_1c_ can simplify the diagnosis of diabetes, as it is a non-fasting test and it can be combined with fasting glucose to make a diagnosis at a single clinic visit [28]. However, in many populations, the 48 mmol/mol (6.5%) HbA_1c_ threshold is more specific and captures a smaller segment of the population as compared with fasting plasma glucose ≥ 7.0 mmol/l (126 mg/dl); thus, a large-scale shift to screening and diagnosis with HbA_1c_ could mean reduced diabetes detection (and incidence) in those tested this way. On the other hand, increased testing could increase diagnoses if it is easier to use HbA_1c_ and fasting glucose in combination (or HbA_1c_ alone for screening) than two consecutive fasting plasma glucose tests. Trends in actual screening and diagnostic practices within healthcare systems are not well documented.

The proportion of those remaining undiagnosed in the total population with diabetes varies substantially globally and influences the denominators for rates of complications and mortality [1, 29]. For example, estimates from the USA [30], Mexico City [31] and cities in South Asia [32] report that 15%, 30% and 26%, respectively, of adults with diabetes are undiagnosed. The proportion of individuals that are undiagnosed might not be a very sensitive indicator of population detection, especially if diabetes prevalence is increasing. More direct population-level information on diabetes testing and how diabetes is identified in practice (e.g. using glucose tests, HbA_1c_ or a combination thereof) is needed to improve our understanding of how diagnostic practices have influenced trends in diabetes burden [28, 30, 33].

The clinical characteristics of the population with diabetes over time could have also influenced trends in complications and mortality rates. The burden of co-existing risk factors (e.g. hypertension and high cholesterol), BMI, use of medications (e.g. angiotensin-modifying agents, statins) and achieving risk factor targets (e.g. HbA_1c_< 53 mmol/mol [7.0%], not smoking) are all relevant. Studies in HIC settings [21, 34, 35] have documented increases in achievement of diabetes care goals, with recent declines noted, especially in older adults [22]. Meanwhile, a few studies in LMICs have noted stagnant [32] or worsening cardiometabolic indicators in adults with known diabetes. The widespread use of statins and BP-lowering therapies and reductions in tobacco use have also occurred in the general population without diabetes, resulting in flattening or reductions in cholesterol levels and BP worldwide [36, 37]. In some countries, like in the USA, those with diabetes have still benefited more than the general population in terms of cardiometabolic profile improvements [38]. Detection earlier in the natural history of diabetes may have led to earlier care initiation and therefore lowered rates of complications and mortality in those with diabetes; however, there are few data quantifying these influences.

To understand the potential impact of earlier detection of diabetes, we can examine trends in retinopathy, which can be detected non-invasively using fundus photography, as an indirect indicator of the population-level effects of screening for diabetes. Data from the 1970s and 1980s in the UK suggested a high prevalence of retinopathy (e.g. 36%) among adults with newly diagnosed type 2 diabetes [39]. More recent data from different but comparable populations suggest a much lower prevalence of retinopathy at the time of diabetes onset: 13% with retinopathy in a large European study (2007–2008), which included milder forms of the disease [40]; 13% in a Danish cohort of individuals with incident diabetes (2010–2016) [41]; 18% in a cohort from Hong Kong (2006–2009) [42]; and 12% in a national survey in the USA (1999–2018) [43]. The decrease in prevalence of retinopathy at diabetes diagnosis between these studies could be an indication that diabetes is being identified earlier in the disease process.

### Mortality

The preferred approach to estimating the composition and contribution of diabetes to mortality is through using population registries and other administrative datasets or epidemiological cohorts to identify a denominator of people with diabetes (whether diagnosed, undiagnosed or both). Via surveillance efforts, mortality and causes of death can be directly evaluated in the population. These data can be used to calculate annual death rates among adults with diabetes as well as the excess risk vs those without diabetes. Published estimates using this approach are limited to North America, Europe, high-income Asian countries and Australia; there are no comparable data in LMICs.

Mortality rates from epidemiological cohorts and diabetes registries can be influenced by screening and diagnostic practices. That is, mortality improvements will be observed if diabetes is captured earlier in the disease process over time. Thus, caution is advised when trying to infer whether mortality declines are due to interventions, programmes or policy, as a larger and/or healthier denominator of people with diabetes can also contribute to declining trends. Trends in excess mortality due to diabetes (i.e. the ratio of mortality rates in people with diabetes over those without diabetes) may be helpful in identifying whether the trends in the diabetes population are occurring faster [11], slower or at the same pace [13], as declines in mortality rates have also been noted in general populations without diabetes.

## How case definitions of numerators can influence rates

The ways in which diabetes complications or mortality are defined in the numerators of published rates over time also influences our understanding of trends.

### Complications

Diabetes complications are routinely identified in healthcare data from information documented in electronic medical records or administrative claims to nationalised health payers or private insurance companies. The potential pitfalls of these data are that case definitions vary; they are reliant on coding by clinicians, which differs in detail, accuracy and reliability. Coding practices are also affected by national or state policies, regulations and incentives [44]. Some datasets permit, while others restrict, linkage with other datasets or linkage from year to year. This influences the interpretation of estimates: in other words, are these the same individuals with multiple visits and discharges for the same reason or are these many individuals with single episodes of care?

There is also variation in how diabetes complications are defined, both by the clinician coding their diagnoses and by the analysts generating estimates. For example, acute coronary events may be defined by any combination of clinical symptoms, elevated biochemical markers (e.g. troponins), ECG abnormalities, angiography and/or imaging. Sensitivity analyses can help assess the robustness of estimates when using a range of definitions [45], but greater standardisation in how diabetes complications are defined from clinical and administrative data would help ensure comparability of data across regions and globally.

A major concern for LMICs is that administrative data are often not used to estimate diabetes-related complications rates. This is often due to a lack of data availability, representativeness, access, timeliness, usability and/or capacity to process and analyse administrative and clinical data. As a result, in LMICs, most estimates of diabetes complications are from surveys in which outcomes are self-reported or modelled using transition probabilities (i.e. relative risks of complications) from HIC data.

### Mortality

As mentioned earlier, one approach to estimating mortality rates is based on death certificates with diabetes coded as an underlying or contributing cause. This is challenging as most deaths in people with diabetes are not due to a direct diabetes-specific cause such as diabetic ketoacidosis or hyperglycaemic hyperosmolar syndrome. Coding of causes of death varies by setting, time and population because of differences in awareness or subjective opinions about the role of diabetes in death. As such, there are concerns about inaccurate coding of diabetes as a cause of death. There may also be systematic differences across socioeconomic status and race/ethnicity in how deaths are coded. It is unclear how frequently coding practices and misclassification of diabetes-related deaths influence diabetes mortality rates. Using diabetes as an underlying cause of death to estimate mortality may also be influenced by diabetes prevalence and diabetes awareness. Studies that use national vital statistics generally report increasing diabetes mortality rates over time. This might explain why mortality rates in LMICs were increasing: the observed trends may represent a problematic method of estimation and increases in diabetes prevalence and not higher diabetes-related mortality per se.

Because of differences in how mortality rates are typically estimated in HICs and LMICs, the WHO and IDF estimate deaths by using models that are based on the prevalence of diabetes and age-specific relative risks of death associated with diabetes. Recent analyses from the IDF indicate that 7%, 12% and 10% of deaths in low-, middle- and high-income countries, respectively, are due to diabetes [46]. This approach is more generalisable across populations since both the denominators and numerators have biases. With regard to relative risks, we observed that HICs have experienced a narrowing of excess risk of complications and mortality conferred by diabetes compared with those without diabetes; there were no comparable data available from LMICs.

## Conclusions and recommendations

Over the past two decades, annual rates of type 2 diabetes-related vascular complications and mortality have been declining in HICs, with persistently high burdens experienced by certain underrepresented and indigenous race/ethnic population subgroups. There were no data for renal, ophthalmic, hepatic or neurological disorders associated with diabetes. Surveillance data for LMICs were either available in few countries or not comparable with data from HICs; this is a critical knowledge gap that must be addressed. If reductions in mortality in people with diabetes become more widespread and are coupled with growth in diabetes prevalence, it is likely that the lifetime accumulation of type 2 diabetes complications and morbidities will grow worldwide [47–49]. It is also possible that with better treatments and management of cardiovascular risk factors, a variety of non-vascular complications for which diabetes is a risk factor (e.g. dementia or infections such as cellulitis and pneumonia) will become relatively more important in individuals with diabetes.

To improve measurement and interpretation of trends in type 2 diabetes complications and mortality rates worldwide, we offer several recommendations (Table 3). First, stronger surveillance infrastructure in LMICs is needed. A comprehensive approach would involve establishing population-level registries that can be linked to hospital discharge data (for complications) and vital statistics data (for cause-specific and excess mortality estimation). This will require governmental investments and efforts by national statistics bureaus to improve the completeness of death certification and cause-of-death assessments and might be especially challenging in rural parts of the world where most deaths occur in non-healthcare settings. Ensuring that registries have a longitudinal component assures a population-based cohort infrastructure that is less subject to biases. That said, in resource-constrained settings, investing in epidemiological cohorts may be an alternative that offers valuable information. However, most cohorts are subject to selection bias and may require periodic expansion (e.g. age period birth cohorts) and sustained investments to provide some semblance of population monitoring and trends.

Second, standardised epidemiological approaches to defining type 1 and type 2 diabetes are needed that balance feasibility, accuracy and precision. This would involve documenting the type and timing (simultaneous or consecutive) of diabetes testing, coding of medications and algorithms that help distinguish diabetes subtypes. This will also open opportunities for more research into types of diabetes testing that are commonly implemented in practice and if and how these influence detection and related care and interventions that follow. Standardising epidemiological definitions of diabetes complications is also important.

Third, focused capacity strengthening is needed in LMICs to build and sustain data management and analytical expertise. This can contribute to more harmonised and comparable estimates of diabetes burdens globally as well as provide opportunities to evaluate the impacts of detection and prevention and management policies and programmes locally and nationally.

Fourth, we propose the development, wider dissemination and implementation of guidelines regarding surveillance reporting; the minimum elements that should be included in the published literature can elevate the credibility and comparability of data globally. A recent guide from the IDF [50] regarding conduct of epidemiological surveys for diabetes addresses reporting standards and offers an exemplar.

Together, these recommendations can advance interpretation and use of international trend data for monitoring and intervention in type 2 diabetes complications and mortality rates.

## Figures and Tables

**Table 1:** Recent (2015 to 2021) publications with population-based data regarding trends in diabetes complications

Country	Study	Years of data	Data	Denominator	Numerator	Findings

USA	captioni [51]	2008–2018	Veterans Affairs database	Veterans (6,493,14–1)	Incidence of LEA	Increased overall (12.9 to 18.1 per 10,000 individuals) but declined in women 62% of the increase was in toe amputations
USA	An [52]	2003–2014	Kaiser Permanente database	Incident T2DM cases (135,199)	Incidence of 13 complications and all-cause mortality	5-year incidence rates declined over time Neuropathy, CKD and CVD were the most common complications
Spain	López-de-Andrés [53]	2001–2018	National hospital discharge database	People with DM	UTI hospitalisation and in-hospital mortality	From 2001–2003 to 2016–2018 admissions per 100,00 individuals increased from 290.8 to 568.5 for DM and 74.8 to 144.0 for non-DM In-hospital mortality declined over time
Spain	Orozco-Beltrán [54]	2005–2015	National hospital discharge database	People with DM	Hospitalisation due to hypoglycaemia and mortality	Admissions per 100,000 individuals decreased from 21.5 to 13.2 in women and from 30.2 to 23.7 in men Mortality (per 100,000 individuals) declined from 8.6 to 4.1 in women and from 9.4 to 6.4 in men
Portugal	Ramalho [55]	2016–2017	National quality improvement registry	People with DM	Preventable hospitalisations	Decreased from 79 to 65.2 per 100,000 individuals
South Korea	Park [56]	2006–2015	National health insurance database	People with DM	Hospitalisation due to vascular complications and mortality	CVD events declined; hospitalisations due to CHF (per 10,000 individuals) increased from 124 to 161 in women and from 72 to 146 in men; hospitalisations for PAD (per 10,000 individuals) increased from 19 to 35 in women and from 39 to 55 in men Mortality from cancers, CVD, DM and HTN declined but mortality from pneumonia increased
South Korea	You [57]	2004–2013	National health insurance database	Population	Hospitalisation due to hyperglycaemia and in-hospital mortality	2004–2006: increased (1.8 to 2.6 per 1000 individuals) 2007–2013: decreased (2.5 to 2.2 per 1000 individuals) Mortality declined
South Korea	Kim [58]	2011–2016	National health insurance database	People with diabetic foot	LEA and revascularisation	Total LEAs increased with flat/declining major amputations; revascularisation interventions increased
Hong Kong	Wu [59]	2001–2016	Electronic medical record diabetes registry	People with DM (770,078)	Hospitalisation for LEA	Decreased (per 10,000 individuals) for minor LEAs (from 14.0 to 7.2 in men [−48.6%] and from 7.9 to 3.2 in women [−59.5%]) and major LEAs (from 19.5 to 4.3 in men [−77.9%] and from 11.6 to 2.4 in women [−79.3%]) Similar findings for newly diagnosed DM and T1DM
Taiwan	Lin [60]	2007–2014	National health insurance database	People with T2DM	Diabetic foot complications (ulcers, infections, gangrene, PAD hospitalisation)	Decreased LEAs (2.9 to 2.1 per 1000 individuals) Major LEAs declined from 56.2% to 47.4% of all LEAs
Brazil	Florêncio [61]	2008–2019	National hospital registry	Population	Hospitalisation related to DM	Increased hospitalisations, higher in female sex; variation in mortality by region

The literature included is composed of articles that reported data with international, national or at least subnational coverage and data sources such as registries or administrative/discharge records. This table does not include publications up to 2015 and is intended as an update to prior reviews [3]

CHF, congestive heart failure; CKD, chronic kidney disease; DM, diabetes mellitus; HTN, hypertension; LEA, lower-extremity amputation; PAD, peripheral arterial disease; T1DM, type 1 diabetes mellitus; T2DM, type 2 diabetes mellitus

**Table 2:** Recent (2015 to 2021) publications with population-based data regarding trends in mortality in adults with diabetes

Country	Study	Year	Data	Denominator	Numerator	Findings

Brazil	Malhão [8]	1980–2012	National vital statistics registry	Population	Age-standardised mortality	Increased (per 100,000 individuals) from 20.8 to 47.6 in men and from 28.7 to 47.2 in women Largest increases were seen up to 2003–2005, then plateaued
Brazil	Klafke [62]	1991–2010	National vital statistics registry	Population	Age-standardised mortality (all-cause and due to acute complications)	Decreased from 8.4 to 2.5 per 100,000 individuals
Colombia	Chaparro-Narváez [63]	1979–2017	National vital statistics registry	Population	Age-standardised mortality	1979–1999: increased (per 100,000 individuals) from 13.2 to 26.6 in women and from 10.1 to 22.7 in men 1999–2017: decreased (per 100,000 individuals) from 2.6 to 15.4 in women and from 22.7 to 15.9 in men
Argentina	Hernández [64]	1990–2013	National vital statistics registry	Population	Age-standardised mortality	1990–2001: increased 2002–2013: decreased Greater declines in women Higher mortality over age 50
Ghana	Sarfo-Kantanka [10]	1983–2014	Tertiary referral hospital (central Ghana)	People with DM (11,414)	In-hospital mortality	Increased from 7.6 to 30.0 per 1000 deaths
South Africa	Nojilana [9]	1997–2010	National vital statistics registry	Deaths in 2010 (594,071)	Cause-specific mortality	Increased to 52 per 100,000 deaths Lower for White vs other groups
UK	Pearson-Stuttard [13]	2011–2018	National primary care database	People with DM (313,907)	Age-standardised mortality (all-cause and DM-specific)	Decline in all-cause mortality in those with DM (31–32%); similar decline in non-DM Cause-specific declines except for dementia and liver disease
USA	Gregg [11]	1988–1994 to 2010–2015	National surveys linked to vital statistics	People with and without DM	Age-standardised mortality (all-cause and DM-specific)	All-cause mortality (per 1000 person-years) declined from 23.1 to 15.2 More marked declines for vascular, then cancer deaths No decline in those aged <45 years
China	Li [65]	2003–2012	National vital statistics registry	Population	Age-standardised mortality	Decreasing More marked in urban populations
Hong Kong	Wu [12]	2001–2016	Electronic medical record diabetes registry	People with DM (390,071 men, 380,007 women)	Age-standardised mortality (all-cause and DM-specific)	All-cause mortality declined (per 100,000 individuals) from 3.3 to 1.7 in women and from 2.8 to 1.5 in men No decline in those aged <45 years
Taiwan	Li [66]	2005–2014	National health insurance linked to vital statistics	People with DM	Age-standardised mortality (all-cause and DM-specific)	All-cause mortality declined (per 100,000 individuals) from 3.1 to 2.7 in women and from 3.8 to 3.3 in men Shorter life expectancy with earlier-onset DM
Australia	Sacre [14]	2002–2014	National diabetes registry	People with T2DM (1,268,018)	Age-standardised mortality (all-cause and DM-specific)	Declines of 1.3–2.2% points per year Declines more pronounced in middle and older ages All-cause, CVD and cancer deaths declined Pneumonia mortality remained stable
New Zealand	Yu [16]	1994–2018	National primary care database	People with T2DM (45,072)	Age-standardised mortality (all-cause and DM-specific)	Increased (per 1000 person-years) from 12.6 before 1998 to 19.4 in 1999–2003, and then decreased to 9.9 per 1000 person-years in 2014–2018
Global	Ling [6]	2000–2016	WHO mortality database	People with T1DM, T2DM or other DM from 108 countries (7,108,145 deaths)	Mortality rates due to renal, ophthalmic, neurological and peripheral circulatory complications	Increased from 46.0 to 60.2 per 100,000 individuals (30.8%) in both men and women Increased in T2DM and decreased in T1DM Higher for renal, neurological and peripheral circulatory complications Increased in all except Asia and South America (declined)
Global	Zaccardi [7]	2000–2014	WHO mortality database	People with DM	Total and hypoglycaemia-related mortality	Increases (per 100,000 individuals) in total (from 912.5 to 1018.8) and hypoglycaemia-related deaths (from 654 to 1248) Lowest and declining rates in Europe, USA, Canada, Japan, NZ and Australia

The literature included is composed of articles that reported data with international, national or at least subnational coverage and data sources such as registries or administrative/discharge records. This table does not include publications up to 2015 and is intended as an update to prior reviews [3]

DM, diabetes mellitus; NZ, New Zealand; T1DM, type 1 diabetes mellitus; T2DM, type 2 diabetes mellitus

**Table 3:** Recommendations to improve estimation and interpretation of diabetes burdens

Category	Benefits

Data infrastructure	
Enhance vital statistics coverage	Decreases biases related to where deaths occur
Enable linkage between community, healthcare and vital registration data systems	Increases validity of reported events
Denominators and numerators	
Validate consensus definitions for diabetes and disseminate	Improves understanding of screening and diagnostic practices and their influence on rates
Expand and standardise routine collection and use of data from healthcare settings	Adds more credible biochemical and clinician-coded indicators to self-reported data
Develop classification structure for diabetes complications (traditional, emerging and other comorbidities)	Elevates importance of non-fatal and non-traditional diabetes complications that affect quality of life
Establish denominators based on standardised definitions	Permits credible comparison of trends within and across countries
Analysis	
Expand capacity to manage data and analyses (especially in LMICs)	Improves surveillance of diabetes burdens and impacts of policies and programmes
Reporting	
Standardise chronic disease surveillance reporting through checklists that recommend providing critical contextual information regarding case definitions and how these are operationalised in the data	Helps analysts and users of data to contextualise and compare the findings

## Key recommendations

- Increase LMIC data
- Standardise definitions
- Strengthen analytical capacity
- Encourage use of reporting guidelines

## Abbreviations

HIC: High-income country
LMIC: Low- and middle-income country
